# Impact of External Environmental Dimensions on Financial Performance of Major Teaching Hospitals in the U.S.

**DOI:** 10.3390/healthcare9081069

**Published:** 2021-08-20

**Authors:** Karima Lalani, Lee Revere, Wenyaw Chan, Tiffany Champagne-Langabeer, Jennifer Tektiridis, James Langabeer

**Affiliations:** 1 Center for Health Systems Analytics, School of Biomedical Informatics, The University of Texas Health Science Center at Houston (UTHealth), Houston, TX 77030, USA; Karima.Lalani@uth.tmc.edu (K.L.); Tiffany.Champagne@uth.tmc.edu (T.C.-L.); 2 Department of Health Services Research, Management and Policy, College of Public Health and Health Professions, University of Florida, Gainesville, FL 32610, USA; FRevere@UFL.edu; 3 School of Public Health, The University of Texas Health Science Center at Houston (UTHealth), Houston, TX 77030, USA; Wenyaw.Chan@uth.tmc.edu (W.C.); Jennifer.H.Tektiridis@uth.tmc.edu (J.T.)

**Keywords:** teaching hospital, financial performance, resource-dependence theory

## Abstract

Teaching hospitals have a unique mission to not only deliver graduate medical education but to also provide both inpatient and ambulatory care and to conduct clinical medical research; therefore, they are under constant financial pressure, and it is important to explore what types of external environmental components affect their financial performance. This study examined if there is an association between the short-term and long-term financial performance of major teaching hospitals in the United States and the external environmental dimensions, as measured by the Resource Dependence Theory. Data for 226 major teaching hospitals spanning 46 states were analyzed. The dependent variable for short-term financial performance was days cash on hand, and dependent variable for long-term financial performance was return on assets, both an average of most recently available 4-year data (2014–2017). Utilizing linear regression model, results showed significance between outpatient revenue and days cash on hand as well as significant relationship between population of the metropolitan statistical area, unemployment rate of the metropolitan statistical area, and teaching hospital’s return on assets. Additionally, system membership, type of ownership/control, and teaching intensity also showed significant association with return on assets. By comprehensively examining all major teaching hospitals in the U.S. and analyzing the association between their short-term and long-term financial performance and external environmental dimensions, based upon Resource Dependence Theory, we found that by offering diverse outpatient services and novel delivery options, administrators of teaching hospitals may be able to increase organizational liquidity.

## 1. Introduction

### 1.1. Background

Health care spending continues to be higher in the U.S. as compared to other developed nations, and the total health care spending as a percentage of U.S. Gross Domestic Product (GDP) has steadily increased since 1970. In 2016, the U.S. spent almost 18% of its GDP on health care [1], and given the unsustainable rise in health care costs, hospitals continue to face various pressures to maximize their efficiency. 

Teaching hospitals, also known as academic hospitals, are complex healthcare entities whose three-pronged mission is to deliver graduate medical education; to conduct medical and clinical research; and to provide both inpatient and ambulatory care [2]. All hospitals in the U.S. continue to face pressures, both financial and non-financial, to improve their quality and efficiency, but teaching hospitals in particular have to juggle not only these pressures but also their mission to provide care, conduct research, and deliver graduate medical education. This research study set out to understand if there is an association between the external environmental dimensions, as measured by the Resource Dependence Theory, and the short-term and long-term financial performance of all major teaching hospitals in the United States.

### 1.2. Public Health Significance

From a public health perspective, teaching hospitals are also an integral part of the healthcare ecosystem of their respective communities; not only do they provide graduate medical education, but they also treat the sickest patients due to the fact that they conduct clinical and medical research, and they treat the neediest of patients as well [3]. Additionally, teaching hospitals account for 21% of all hospital beds in the U.S. [4]; plus, they carry a large burden of charity care for the poorest of patients by caring for almost 40% of the uninsured, and they account for almost 33% of national health-related funds for research [5]. 

Teaching hospitals also produce approximately 22,000 medical school graduates annually since they are the dominant providers of graduate medical education (GME) [5]. Additionally, they also graduate about 15,000 nurses and 6000 public health professionals [5]. Furthermore, teaching hospitals contributed approximately $380 billion in value to the U.S. economy and supported over 6 million jobs in 2017 [6].

### 1.3. Literature Review

#### 1.3.1. Overview of Teaching Hospitals

As the clinical enterprise of an academic health center, teaching hospitals have a unique, three-pronged mission to deliver not only graduate medical education but also to provide both inpatient and ambulatory care and conduct clinical and medical research [2]. In order to conduct graduate medical education, the training of medical school graduates is organized around the day-to-day operations of a teaching hospital [2]. The resident physicians treat patients under the supervision of faculty physicians; thus, both the patient care and the medical education takes place simultaneously in a teaching hospital.

Additionally, teaching hospitals conduct a wide range of clinical and medical research. They are the primary centers of research, and over the last several decades, new approaches to diagnosis and prevention as well as medical breakthroughs and innovations have been pioneered at teaching hospitals [6]. Given the fact that the teaching hospitals are fulfilling their unique missions and advancing clinical and medical research, it is important to examine which factors and aspects are affecting the teaching hospitals’ financial performance and what types of strategies ought to be examined and implemented by the teaching hospitals’ administration and leadership.

#### 1.3.2. Empirical Studies on Hospital Financial Performance since the 1980s

Several empirical studies have been conducted over the last few decades about hospitals and their financial performance; however, studies that have focused solely on teaching hospitals’ financial performance have been conducted with less frequency. A study of 64 teaching hospitals in the 1980s analyzed the impact of state-level environment on hospitals’ financial performance [7]. A study about teaching hospitals from 1986 concluded that although all teaching hospitals face constant financial pressures, those teaching hospitals that are under government control are in a more precarious financial position as compared to the non-municipal teaching hospitals [8].

In the 1990s, it was noted by the Council on Graduate Medical Education that the financial situation of major teaching hospitals in the U.S. had deteriorated, and the major teaching hospitals had the lowest margins in the hospital industry [9]. A study in 2000 utilized activity-based cost analysis at a teaching hospital to enhance the financial performance of a clinical department [10]. Another study from early 2000s analyzed cost inefficiencies in 211 major teaching hospitals [11]. Results from a study done on a sample of 50 major teaching hospitals in 2004 found that nearly one out of six major teaching hospital in the sample was near immediate bankruptcy [12]. A study from 2011 examined data for only 103 teaching hospitals and concluded that the hospitals with low cash flow also tend to have higher operating losses and low financial performance [13]. A study conducted in 2013 analyzed data from 117 teaching hospitals and concluded that large teaching hospitals located in urban areas were more likely to have higher fixed costs and lower variable costs [14].

A study conducted in 2015 looked at all nonfederal, acute-care public hospitals in the U.S. and concluded that the teaching hospitals should undergo either privatization, complete restructuring, or other strategic changes to overcome their financial challenges [15]. A study conducted in 2017 looked at the major teaching hospitals in only the 20 largest U.S. cities and found that the economic status of the surrounding community, the hospital’s size, and teaching intensity were more important than operational efficiency [16]. A qualitative study on a sample of 20 major teaching hospitals found that the leadership of high-performing major teaching hospitals foster a vision and mission for their teaching hospital to deliver consistent, high quality of care to their patients and communities [17].

#### 1.3.3. Theoretical Framework

Teaching hospitals are organizations under constant financial pressure, and their financial performance is affected by various environmental factors. Resource-dependence theory (RDT) posits that cues from the availability or scarcity of external resources, such as a skilled workforce, a stable environment, or the level of organizational complexity, may provide a strategic direction for administrators to employ to ensure a hospital’s long-term financial performance and survival [18]. An organization’s environment, as in the case of a teaching hospital, consists of other entities from which it procures resources and to which it sells products and services. Because an organization’s possession and control of key resources imply power, organizations must adopt various strategies to acquire and control resources, reduce dependencies, and increase their power. Scarce resources motivate managers to act in ways that will secure those resources and reduce uncertainty [19]. 

No recent studies have been comprehensively conducted for all major teaching hospitals in the United States, and according to the American Association of Medical Colleges, there are close to 300 major teaching hospitals in the U.S. [20]. Only a few studies have examined the impact of a teaching hospital’s strategy and operations on its financial performance, and these studies were conducted decades ago [21,22]. This study set out to understand and seek answers to the question of whether there is an association between the external environmental dimensions and the short-term financial performance as well as the long-term financial performance of all major teaching hospitals in the United States.

## 2. Materials and Methods

### 2.1. Study Design, Study Sample 

This study was a retrospective, cross-sectional, observational study, using publicly available secondary data from 2014 to 2017. The study population included all short-term, acute-care hospitals in U.S., using the Association of American Medical Colleges’ Council of Teaching Hospitals and Health Systems (COTH) criteria for major teaching hospitals [20]. Membership to the Council of Teaching Hospitals and Health Systems (COTH) is for those teaching hospitals that meet the following criteria: (1) have a documented affiliation agreement with an accredited U.S. medical school and (2) have minimum of four approved, active residency programs, including at least two approved residency programs in medicine, surgery, pediatrics, family practice, psychiatry, or obstetrics/gynecology [23]. 

Empirically, the findings of a study on teaching hospitals conducted in early 1990s suggested that major academic hospitals should be studied as a group separately from other non-major teaching and non-teaching hospitals when measuring the hospital’s performance [24]. This study’s population, based upon the AAMC data, consisted of 226 hospitals in 46 states, representing 80% of all major teaching hospitals in the U.S. Total COTH members as of March 2019 were 282 teaching hospitals. This study excluded 6 specialty hospitals, 17 children’s hospitals, and 33 Veterans Administration (VA) hospitals. The VA hospitals are under the purview and financing of Veterans Health Administration, under U.S. Department of Veterans Affairs [25]. Specialty hospitals do not have comparative patient populations as other general, short-term acute-care hospitals and treat less complex and more profitable cases [26]; thus, these hospitals were also excluded from this study. The states where these major teaching hospitals are located are 46 states plus the District of Columbia. Four states, Alaska, Hawaii, Idaho, and Wyoming, did not have a COTH member hospital as of March 2019.

### 2.2. Operationalization of Study Variables

#### 2.2.1. Operationalization of Dependent Variables 

In order to measure short-term financial performance, aim 1 of this study, the dependent measure was the financial measure of liquidity called days cash on hand. In financial literature, this measure of liquidity is calculated as:

*Days Cash on Hand (all sources) = (Cash + Market Securities + Short-term Investments) ÷ (Total operating expenses − Depreciation)/365

This financial measure illustrates the number of days that an organization can continue to pay its cash obligations if no new cash resources became available. High positive values of days cash on hand imply that an organization has high liquidity, and the organization can then be viewed favorably by creditors [27,28]. For the purposes of this study, the calculation of “Days Cash on Hand—All Sources” has been used and will be referred to as “days cash on hand” throughout this study. 

In order to measure long-term profitability, aim 2 of this study, the dependent measure was the financial measure of profitability called return on assets” (ROA). In financial literature, this measure of profitability is calculated as:

Return on Assets (ROA) = (Net Income/Total Assets) × 100

This financial measure of profitability illustrates how an organization can use its assets to generate income; for example, if an organization has a return on assets of 12%, then it means that each dollar invested in total assets produces 12 cents in profits. A high ROA denotes that the organization’s assets are financially productive [27,28]. In the healthcare context, a hospital’s return on assets is considered a key indicator that can reflect the hospital’s ability to fulfill its current operational funding as well as its ability to take care of funding any future increases in assets [29].

#### 2.2.2. Operationalization of Independent Variables

Empirical studies in the healthcare management realm have posited how the RDT dimensions of munificence, uncertainty, and complexity can be operationalized. The independent variables of the income per capita, population per capita, as well as the hospital’s urban location have been empirically used to operationalize the RDT dimension of munificence [30,31]. Uncertainty can be operationalized through the independent variable of unemployment rate change of the metropolitan statistical area [30,31] because high rates of unemployment in an area produce uncertainty for the population. 

The RDT dimension of complexity can be operationalized through the independent variable called the Herfindahl–Hirschman Index (HHI), which is a commonly accepted measure of market competition [30,31]. HHI measures the amount of competition among firms in a particular market and is the sum of all facilities’ squared market share [32]. HHI in this study was calculated based upon the metropolitan statistical area (MSA) where the major teaching hospital is located instead of at the county level since major teaching hospitals serve a broader community in the MSA [32]. The U.S. Department of Justice considers markets in which the HHI is between 1500 and 2500 points to be moderately concentrated and markets in which HHI is higher than 2500 points to be highly concentrated [33].

#### 2.2.3. Operationalization of Control Variables 

Research studies in the healthcare management literature have utilized several different control variables. For the purposes of this study, seven control variables were used, namely system membership, type of ownership or control of hospital; geographic region; number of beds; teaching intensity, measured by number of medical residents; case mix index; and percentage of outpatient revenue. For all the study measures, their data sources and definitions are summarized in Table 1.

### 2.3. Ethical Considerations

This study was approved by the University of Texas Health Science Center’s Institutional Review Board as exempt. No human subjects were used or considered for this study, and no protected health information or personal information was used.

### 2.4. Statistical Analysis 

Descriptive statistics were used to assess each variable, including means and standard deviations for normally distributed data, and differences among independent variables were explored using ANOVA and chi-square analysis. Linear regression was used to analyze both long-term and short-term profitability, using factors with significant univariate results. 

Nonlinearities in regression analysis are incorporated by transforming the dependent variable in logarithmic form [43]. All the variables, including the independent variables, were graphically inspected for normality before beginning the regression analysis as well as by analysis of histograms, kernel density plots, normal quantile plots, and normal probability plots in STATA-14 [44]. The two dependent variables, days cash on hand and return on assets, did not meet the criteria of normality after examination of their respective plots; hence, the ladder command in STATA-14 was utilized to assist in the transformation of the two dependent variables, and the logarithmic transformation of the two dependent variables was used for this study. The independent variables in this study did not warrant logarithmic transformations and thus were not transformed. 

Once the regression models were run for both aims of this study, additional regression diagnostics were analyzed to assess the goodness of fit of the regression models. The predict command in STATA-14 was utilized to create residuals, and then kernel density plots, quantile normal plots, and normal probability plots were used to check the normality of the residuals [44]. Additionally, since STATA-14 assumes homoskedastic standard errors by default, the two models were adjusted to account for heteroskedasticity, by using the option of robust in STATA-14 [44] after the Breusch–Pagan/Cook–Weisberg test for heteroskedasticity detected heteroskedasticity in both regression models [44]. STATA-14 commands were also used to detect any multicollinearity in the two models. When multicollinearity is present, the standard errors in the regression model may be inflated [45]; therefore, the command VIF (variance inflation factor) was used to detect multicollinearity since any value variable that has VIF value of greater than 10 would require further investigation [46]. None of the variables in both regression models for this study had any VIF value of 10 or greater. Regression model-specification tests were also conducted in STATA-14 for both regression models of this study. The two model-specification tests were the linktest and the omitted variable test in STATA-14 [44]. The linktest for both models generated *p*-values of the squared prediction variable, _hatsq, to be greater than 0.98, and the omitted variable test was not significant and confirmed that no variables were omitted in both regression models.

## 3. Results

Data from 226 major teaching hospitals were included in the analysis. Table 2 below provides the descriptive statistics for all variables used in this study. 

The overall results for these major teaching hospitals were remarkable. For the short-term financial performance of major teaching hospitals, results of the regression model showed an increase in outpatient revenue to be significantly associated with days cash on hand. For the long-term financial performance of major teaching hospitals, the study showed significant relationships between the munificence and uncertainty dimensions of the teaching hospital’s external environment and its return on assets. Additionally, system membership, type of ownership/control, and teaching intensity also showed significant associations with long-term financial performance.

Regression results of the study showed no significant relationship between the short-term financial performance measured by days cash on hand all sources and the Resource Dependence Theory’s dimensions of munificence, uncertainty, or complexity of the teaching hospital’s external environment; however, there was significance between short-term financial performance and outpatient revenue, showing that a one-percent increase in outpatient revenue will increase days cash on hand by 12.61% (*p*-value < 0.039). After multivariate controls, the final regression model for days cash on hand showed significance with only one independent variable: outpatient revenue percentage (β = 2.53; *p*-value < 0.039). The regression model results for days cash on hand are provided in Table 3. The R^2^ value of 0.050 for the regression model results indicates that there are other explanatory variables that may explain the relationship with the dependent variable of days cash on hand in this model. In social sciences research, low values of R^2^ are not uncommon [43].

This study also showed a significant relationship between the long-term financial performance, measured by return on assets, and the Resource Dependence Theory’s dimensions of munificence and uncertainty of the teaching hospital’s external environment. There was no significant relationship between long-term financial performance and the complexity of the external environment, measured by the HHI. Additionally, system-affiliated teaching hospitals had 2.05% higher ROA as compared to non-system affiliated teaching hospitals (*p*-value < 0.009). This study’s sample had 193 hospitals that were system-affiliated and 33 hospitals that were not system affiliated. Teaching hospitals under proprietary control had almost 2.51% higher ROA as compared to teaching hospitals under non-profit control (*p*-value < 0.033). Additionally, for every 1-unit increase in teaching intensity (number of residents), the ROA will decrease by 0.99% (*p*-value < 0.047). The regression model results for return on assets are provided in Table 4. The R^2^ value of 0.192 for the regression model results indicates that there are other explanatory variables that may explain the relationship with the dependent variable of return on assets in this model. In social sciences research, low values of R^2^ are not uncommon [43].

## 4. Discussion

Hospitals in the U.S., specifically the teaching hospitals, are facing financial challenges and pressures and will continue to do so as the momentum in the U.S. moves towards providing more value-based health services that will keep populations healthy. This study explored whether there is an association between the short-term and long-term financial performance of major teaching hospitals and the external environmental dimensions based upon the Resource Dependence Theory framework. Based upon the literature review, this study is the first of its kind to comprehensively study all major teaching hospitals in the U.S. and their short-term financial performance based upon the financial measure of liquidity, called days cash on hand, and their long-term financial performance based upon the financial measure of profitability, called return on assets, from the perspective of the Resource Dependence Theory. 

The regression analysis of this study found a significant positive association between the measure of liquidity days cash on hand and outpatient revenue of the teaching hospital. This study confirms the finding from a previous study that increasing percentage of outpatient revenue will result in reducing financial difficulty or financial distress of the hospital [41]. Financial distress is the term that is used in financial management literature to refer to those organizations that have difficulties in paying their creditors, employees, and investors [47]. 

Empirical studies in healthcare-management literature have operationalized the dimensions of the Resource Dependence Theory with the variables of per capita income of the MSA, population of the MSA, and the teaching hospital’s location in order to study munificence of the external environment, the unemployment rate change of the MSA to study uncertainty of the external environment, and the Herfindahl–Hirschman Index (HHI) to study the complexity of the external environment. This study’s statistical analysis did not find significance between these specific external environmental components and the short-term liquidity measure chosen for this study. 

Regarding long-term financial performance, this study found the Resource Dependence Theory-based operationalized variables showed significance with the dimensions of munificence and uncertainty of the external environment. The regression results confirm prior findings about significant relationship between hospital’s performance and population of the metropolitan statistical area [32], the level of unemployment rate change [40], system affiliation [41], and teaching intensity [16]. 

Despite the regression results showing negative relationship between the teaching intensity and return on assets, this study is not suggesting that a teaching hospital should reduce their number of residents to achieve long-term profitability. Employing a higher number of graduate medical students will increase labor costs, but it will increase the human resource capacity in the hospital and will allow for greater efficiencies to treat higher volumes of patients [16].

Hospital administrators can analyze their respective service lines and revenue mix to offer more outpatient services to their patients and surrounding community. Furthermore, administrators and managers at major teaching hospitals can explore novel ways of delivering care by utilizing telehealth and other technological innovations. Administrators of teaching hospitals can also consider developing efficiencies in their accounts receivables system to better manage their cash flow and liquidity. 

Another area for administrators of major teaching hospitals to consider is the population of their metropolitan area. This study’s findings showed a negative significant relationship between the level of profitability and an increase in the population of the surrounding metropolitan statistical area. Hospital administrators can analyze their respective metropolitan area’s population growth patterns based upon specific age groups to ensure that the optimal mix and types of services are being offered and rendered that can continue to ensure the desired level of long-term profitability of the teaching hospital. 

Smitherman et al. proposed that rather than the traditional three-pronged mission of teaching hospitals, addressing the social determinants of health should allow teaching hospitals to have a “quadripartite mission” to address social accountability [5]. A number of social science and public health researchers have also concluded that socioeconomic components as well as living conditions now account for over 60% of all chronic disability and premature deaths in the U.S. [5]. Teaching hospitals are in a unique position to take a leadership role in their communities to partner with pertinent stakeholders to improve the health of their local population. 

Another one of this study’s findings was that system-affiliated hospitals have 2.05% higher return on assets, assuming all other variables remain constant, as compared to non-system-affiliated teaching hospitals. This finding is consistent with the assumptions and rationale of Resource Dependence Theory because strategic alliances are one of the ways organizations adopt to increase their control over resources [18]. Administrators at non- system-affiliated hospitals may want to consider evaluating the feasibility of a health system alliance in the turbulent financial environment, either centralized, moderately centralized, or decentralized health system [11] and how it will impact both short-term liquidity and long-term profitability. Another approach towards affiliation can be strengthening the teaching hospital’s access within a geographic region, and mergers, acquisitions, as well as strategic geographic partnerships can also help teaching hospitals to broaden their area of service [48]. 

Regarding the type of ownership and control, this study’s regression results found that teaching hospitals under proprietary control have almost 2.51% higher return on assets as compared to teaching hospitals that are under non-profit control, assuming all other variables remain constant. Type of ownership an organization maintains to reduce its dependence over resources and increase control over the resources in the environment are consistent with the rationale of Resource Dependence Theory [18]. A proprietary, for-profit control of an organization denotes answering to external shareholders and creditors about the organization’s income, profits, or losses as well as operational and leadership trajectories, and hospital administrators will need to assess the long-term organizational strategy before considering changing their ownership type and control. 

Teaching hospitals are also primary centers of research, and over several decades, novel approaches to diagnosis and prevention as well as medical innovations have been pioneered at these hospitals [6]. Translating academic clinical research into patient care improvement and innovative breakthroughs is not an easy task, and in the current turbulent market of shrinking research budgets and financial constraints, a gap exists between this type of clinical research and commercializing it; thus, health care innovation centers have filled this gap recently [49]. Health care innovation centers tend to be partnerships between academic and medical institutions and provide education, mentoring, advising, as well as funding to innovators who want to solve real-world healthcare problems, and teaching hospitals are again positioned to partner with relevant stakeholders to commercialize promising clinical research to improve patient care and invent medical breakthroughs [49]. 

### 4.1. Strengths and Limitations

One of the strengths of this study is that it analyzed all the major teaching hospitals in the U.S. and their cash liquidity and long-term profitability for the years 2014 to 2017. This study provides an observational, cross-sectional analysis of how the major teaching hospitals are faring in the current era of rising healthcare expenditures and financial turbulence. Another strength of this study is that the external environmental dimensions based upon the Resource Dependence Theory were operationalized in this research to explore an association between those external environmental components and both the short-term and long-term financial performance of major teaching hospitals. 

The third strength of this study is that this research also sought to fill gaps in the healthcare management literature about the applicability of Resource Dependence Theory in the specific context of major teaching hospitals. Although Pfeffer and Salancik’s work on Resource Dependence Theory has been studied in healthcare settings, such as hospitals, nursing homes, and medical practices, this study adds to the growing corpus of healthcare studies but with specific focus on major teaching hospitals and the external environment’s impact on their financial performance [18].

As with all research studies, this study also has limitations. One of the limitations is that this study analyzed data for major teaching hospitals in the U.S., i.e., those teaching hospitals in the U.S. that are members of the Council of Teaching Hospitals and Health Systems (COTH); thus, results of this study may not be generalizable to teaching and non-teaching hospitals that are not members of COTH and maybe located in smaller communities, metropolitan areas, and other countries. 

Another limitation of this study is that the data used are not derived from primary sources but from secondary sources; however, publicly available national sources of data were used for this study in order to mitigate the effect of this specific limitation. An additional limitation of this study is that hospitals in the U.S. have different fiscal reporting cycles, and hence, the averages for their reported financial data were taken for this study. 

In addition, there are other types of financial measures that could have been used for this study. The liquidity measure of days cash on hand was chosen for this study to shed light on how many days can major teaching hospitals operate with if no new sources of cash became available to them. Additionally, the long-term financial measure of return on assets was used in this study because this measure is more comprehensive since it considers both the net income and total assets compared to other long-term profitability measures, like operating margin or growth rate in equity [27]. 

Finally, this study was a retrospective, cross-sectional study, providing a snapshot into the liquidity and profitability of major teaching hospitals only in the U.S. for a certain point in time, namely from 2014 to 2017. The findings of this research, therefore, may vary for different time periods as well as for other countries.

### 4.2. Areas of Future Research

This research study can be used as a foundation for multiple future research studies. One area of future research can be expansion of this study’s design based upon the operationalization of Resource Dependence Theory and extending it to all teaching hospitals in the U.S. and not just the major teaching hospitals. A second area of future research can be to expand this study’s theoretical framework for all hospitals, not just the teaching hospitals, in the U.S. and globally. A third area of future research can explore combining multiple organizational management theories, like transaction cost economics and institutional theory, with Resource Dependence Theory and operationalizing them to study financial performance of various types of healthcare facilities. All healthcare settings are operating with varying degrees of uncertainty and complexity in their respective external environments, and future research can shed light upon any interrelationships amongst the strategies used to reduce external environmental dependencies.

Future research can also explore the association between short-term and long-term financial performance of teaching hospitals by using different measures of liquidity, like the current ratio and quick ratio, and different measures of profitability, like growth rate in equity, operating margin, and total margin [27]. Another future research study can examine the impact of specific healthcare technologies, like telehealth and diagnostic imaging, on both the short-term and long-term performance of teaching hospitals.

Quantitative studies are not the only options for future research. This study’s findings can also be utilized for qualitative studies that can look for themes that may emerge from observations and evaluations of certain contexts. Semi-structured or structured interviews of various decision makers and members of leadership team at teaching hospitals can be conducted to ascertain a more holistic understanding of strategies they are using and plan to use in the future to navigate the challenging financial terrain. Additionally, mixed-methods studies can also be conducted based upon this study’s findings, incorporating both quantitative and qualitative research methodologies.

## 5. Conclusions

This study set out to understand if there is an association between the external environmental dimensions and the short-term and long-term financial performance of all major teaching hospitals in the United States, and this study found a significant positive association between the number of days cash on hand and the outpatient revenue of the teaching hospital. This study also confirmed prior findings about significant relationship between hospital’s performance and population of the metropolitan statistical area, the level of unemployment rate change in the metropolitan area; system affiliation, and teaching intensity.

Currently, the U.S. healthcare environment is operating in a state of flux and uncertainty, and with the high level of attention focused on national health expenditures and healthcare organizations, the application of Resource Dependence Theory perspective in research studies of healthcare organizations has become more critical and relevant than ever before. Resource Dependence Theory assumes that organizations will minimize their dependence on other organizations in the environment for the supply of resources, and the organization’s survival will be threatened if it is unable to secure the needed resources. Managers and decision-makers need to continually engage with strategies and innovative organizational alliances and linkages to ensure the organization’s survival, growth, and reduced dependence on resources. This study fills the gaps in the healthcare management body of knowledge about the financial performance of major teaching hospitals in the United States. Results from this study can be used by teaching hospital administrators to further optimize their revenue streams proactively and to continue to be engaged with their local communities to work towards population health improvements.

Despite the challenging healthcare landscape, major teaching hospitals have continued to maintain and fulfill their clinical, academic, and research missions. This study’s findings can help the administrators and decision makers at these institutions to formulate and implement strategies that can increase both their short-term and long-term financial performance. This research suggests that increasing percentage of outpatient revenue can be an important element to consider for the major teaching hospitals to increase their cash liquidity as a component of their approach towards increasing the organization’s liquidity. The unique mission of clinical research, medical education, and patient care are foundations of the exceptional institutions called teaching hospitals. As the confluence of financial, operating, regulatory, and technological changes continue to shape the U.S. healthcare industry, findings and suggestions from this study may help the administrators and leadership of teaching hospitals in the U.S. to analyze and evaluate their existing strategies, and align suggestions towards enhancing their unique mission while ensuring successful short-term and long-term financial performance of their hospitals.

## Figures and Tables

**Table 1:** Study Measures.

Variables	Type of Variable	Related Aim	Unit of Analysis	Data Source	Definition	Literature Reference
***Dependent Variables***						
Days Cash on Hand	Continuous	Short-term financial performance (Aim 1)	Hospital	Medicare Cost Report data from American Hospital Directory database [34]	A measure of company’s liquidity; whether it can meet its payments when they are due.	Gapenski, 2012 [27]
Return on Assets	Continuous	Long-term financial performance (Aim 2)	Hospital	Medicare Cost Report data from American Hospital Directory database [34]	Measures a company’s ability to control expenses and its ability to use its assets to generate revenue.	Gapenski, 2012 [27]
***Independent Variables***						
***Resource Dependence Theory Dimension of Munificence***						
MSA Income per Capita	Continuous	Aim 1 Aim 2	MSA	U.S. Dept of Commerce, Bureau of Economic Analysis [35]	Per capita income of metropolitan statistical area (MSA) where teaching hospital is located.	Ginn, 1992 [36]; Zinn, 1997 [37]
MSA Population	Continuous	Aim 1 Aim 2	MSA	U.S. Census Bureau [38]	Population of the metropolitan statistical area (MSA) where teaching hospital is located.	Balotsky, 2005 [32]
Urban Location	Yes = 1 No = 0	Aim 1 Aim 2	Hospital	Medicare Cost Report data from American Hospital Directory database [34]	Designation of 1 if the hospital is in an urban area; otherwise, designation of 0.	Zinn, 1997 [37]
***Resource Dependence Theory Dimension of Uncertainty***						
Level of Unemployment Rate Change	Continuous	Aim 1 Aim 2	MSA	U.S. Dept of Labor, Bureau of Labor Statistics [39]	Level of unemployment rate change at the metropolitan statistical area (MSA) level.	Kazley, 2007 [40]
***Resource Dependence Theory Dimension of Complexity***						
Herfindahl–Hirschman Index (HHI)	Continuous	Aim 1 Aim 2	MSA	Calculated using Medicare Cost Report data [34]	A measure of market concentration; the amount of competition among firms in a particular market; sum of all facilities’ squared market share.	Balotsky, 2005 [32]
***Control Variables***						
System Membership	Yes = 1 No = 0	Aim 1 Aim 2	Hospital	Hospital Characteristics data from American Hospital Directory database [34]	Denotes a hospital’s membership in a health system.	Langabeer, 2018 [16,41]
Ownership/Control	Categorical (Voluntary Nonprofit; Government; Church; Proprietary)	Aim 1 Aim 2	Hospital	Medicare Cost Report data from American Hospital Directory database [34]	Type of ownership or control of the hospital.	Langabeer, 2018 [16,41]
Geographic Region	Categorical 1 = Region 1 2 = Region 2 3 = Region 3 4 = Region 4	Aim 1 Aim 2	Hospital	U.S. Census Bureau [38]	Hospital’s location in one of four U.S. geographic regions.	Horwitz, 2015 [42]
Number of Beds	Continuous	Aim 1 Aim 2	Hospital	Medicare Cost Report data from American Hospital Directory database [34]	Number of beds in a hospital.	Langabeer, 2018 [16]
Teaching Intensity	Continuous	Aim 1 Aim 2	Hospital	Medicare Cost Report data from American Hospital Directory database [34]	Number of medical residents in a teaching hospital.	Langabeer, 2018 [16]
Case Mix Index	Continuous	Aim 1 Aim 2	Hospital	Medicare Case Mix Index data from American Hospital Directory database [34]	Reflects the clinical complexity and resources needs of all patients in a hospital; more complex case loads are indicated by high case mix index.	Langabeer, 2018 [41]
Outpatient revenue %	Continuous	Aim 1 Aim 2	Hospital	Calculated using Medicare Cost Report data [34]	Percentage of hospital’s total revenue attributed to outpatient services.	Langabeer, 2018 [41]

**Table 2:** Descriptive Statistics and Hospital Characteristics.

Variable	Total
Hospitals, n	226
Days Cash on Hand, mean (SD)	141 (257)
Return on Assets as %, mean (SD)	6.58% (0.1398)
MSA per Capita Income ($ per 10,000), mean (SD)	5.36 (1.24)
MSA Population (in 1,000,000 s), mean (SD)	5.05 (6.17)
MSA Unemployment Rate Change as %, mean (SD)	−0.90% (0.026)
MSA Herfindahl–Hirschman Index (HHI), mean (SD)	1990 (1919)
Number of Beds, mean (SD)	678 (455)
Teaching Intensity, mean (SD)	314 (233)
Case Mix Index, mean (SD)	1.937 (0.262)
Outpatient Revenue %, mean (SD)	44.65% (0.1045)
Urban, n (%)	180 (79.65%)
Rural, n (%)	46 (20.35%)
System Membership	
Yes, n (%)	193 (85.40%)
No, n (%)	33 (14.60%)
Type of Ownership/Control	
Voluntary Non-Profit, n (%)	141 (62.39%)
Church, n (%)	19 (8.41%)
Government, n (%)	54 (23.89%)
Proprietary, n (%)	12 (5.31%)
Geographic Region	
Northeast, n (%)	67 (29.65%)
Midwest, n (%)	54 (23.89%)
South, n (%)	73 (32.30%)
West, n (%)	32 (14.16%)

**Table 3:** Regression Model Results for Study Aim 1, Days Cash on Hand (Dependent Variable).

Variable	Coefficient	*p*-Value	95% C.I.
MSA per Capita Income ($ per 10,000)	0.074	0.321	(−0.073, 0.223)
MSA Population (in 1,000,000s)	0.019	0.349	(−0.021, 0.060)
Urban Location	−0.170	0.537	(−0.713, 0.372)
Unemployment Rate Change	−2.111	0.089	(−4.544, 0.321)
MSA HHI	0.0001	0.1	(−0.00001, 0.00002)
System Membership	−0.170	0.405	(−0.573, 0.232)
Ownership/Control	−0.133	0.756	(−0.973, 0.707)
Geographic Region	−0.308	0.385	(−1.004, 0.389)
Number of Beds	0.0008	0.058	(−0.00003, 0.0017)
Teaching Intensity	−0.0002	0.774	(−0.0016, 0.0012)
Case Mix Index	0.367	0.477	(−0.648, 1.383)
Outpatient Revenue %	2.534	0.039	(0.127, 4.943)
Constant	2.044	0.188	

R^2^ = 0.050.

**Table 4:** Regression Model Results for Study Aim 2: Return on Assets (Dependent Variable).

Variable	Coefficient	*p*-Value	95% C.I.
MSA per Capita Income ($ per 10,000)	−0.010	0.891	(−0.160, 0.139)
MSA Population (in 1,000,000 s)	−0.026	0.041	(−0.051, −0.001)
Urban Location	0.087	0.612	(−0.252, 0.427)
Unemployment Rate Change	−4.626	0.001	(−6.021, −3.230)
MSA HHI	−0.00000179	0.963	(−0.00000786, 0.000075)
System Membership	0.719	0.009	(0.181, 1.258)
Proprietary Control	0.920	0.033	(0.076, 1.764)
Number of Beds	0.000153	0.216	(−0.0000908, 0.0003984)
Teaching Intensity	−0.000764	0.047	(−0.0015, −0.00899)
Case Mix Index	0.482	0.183	(−0.2297, 1.1941)
Outpatient Revenue %	−0.610	0.399	(−2.035, 0.815)
Constant	−4.044	0.001	

R^2^ = 0.192

## Data Availability

This study used publicly available secondary data, and the data sources have been provided in Table 1.
